# Melatonin Promotes Oligodendroglial Maturation of Injured White Matter in Neonatal Rats

**DOI:** 10.1371/journal.pone.0007128

**Published:** 2009-09-22

**Authors:** Paul Olivier, Romain H. Fontaine, Gauthier Loron, Juliette Van Steenwinckel, Valérie Biran, Véronique Massonneau, Angela Kaindl, Jeremie Dalous, Christiane Charriaut-Marlangue, Marie-Stéphane Aigrot, Julien Pansiot, Catherine Verney, Pierre Gressens, Olivier Baud

**Affiliations:** 1 INSERM, AVENIR R05230HS, Hôpital Robert Debré, Paris, France; 2 INSERM, UMR 676, Université Denis Diderot Paris 7, Hôpital Robert Debré, Paris, France; 3 CNRS, UMR 7101, Université Pierre et Marie Curie Paris 6, Paris, France; 4 INSERM, UMRS 975, CNRS UMR 7225, CRICM, Hôpital de la Salpétrière, Paris, France; 5 APHP, Neonatal Intensive Care Unit, Hôpital Robert Debré, Paris, France; 6 PremUP Foundation, Paris, France; Chiba University Center for Forensic Mental Health, Japan

## Abstract

**Objective:**

To investigate the effects of melatonin treatment in a rat model of white matter damage (WMD) in the developing brain. Additionally, we aim to delineate the cellular mechanisms of melatonin effect on the oligodendroglial cell lineage.

**Methods:**

A unilateral ligation of the uterine artery in pregnant rat at the embryonic day 17 induces fetal hypoxia and subsequent growth restriction (GR) in neonatal pups. GR and control pups received a daily intra-peritoneal injection of melatonin from birth to post-natal day (P) 3.

**Results:**

Melatonin administration was associated with a dramatic decrease in microglial activation and astroglial reaction compared to untreated GR pups. At P14, melatonin prevented white matter myelination defects with an increased number of mature oligodendrocytes (APC-immunoreactive) in treated GR pups. Conversely, melatonin was not found to be associated with an increased density of total oligodendrocytes (Olig2-immunoreactive), suggesting that melatonin is able to promote oligodendrocyte maturation but not proliferation. These effects appear to be melatonin-receptor dependent and were reproduced *in vitro*.

**Interpretation:**

These data suggest that melatonin has a strong protective effect on developing damaged white matter through decreased microglial activation and oligodendroglial maturation leading to a normalization of the myelination process. Consequently, melatonin should be a considered as an effective neuroprotective candidate not only in perinatal brain damage but also in inflammatory and demyelinating diseases observed in adults.

## Introduction

Brain injury and subsequent neurodevelopmental disabilities resulting from premature birth are a major public health concern. Indeed, preterm birth survivors can suffer from long term clinical, educational and social problems: 10–15% of infants surviving from very preterm delivery develop cerebral palsy and 50% show adverse neurodevelopmental outcome at 30 months of age. Due to dramatic improvement in perinatal management of high risk preterm neonates, pathological conditions associated with neurological impairment have changed over the past 10 years. Major focal destructive lesions remain serious but have become less common. In contrast, the most prominent neuropathological lesion is diffuse white matter damage (WMD) showing an association of glial injury together with microglial activation and, ultimately, myelination defects.

Many factors are associated with WMD, including: infection, hypoxia, ischemia, endocrine imbalances, genetic factors and growth restriction [1]–[3]. In particular, intra uterine growth retardation (IUGR) has been shown to be associated with both white matter lesions and subsequent neurological impairment [4]–[6] Based on these potential targets, a number of treatments for neonatal brain injury including melatonin have been investigated [7].

The pleiotropic effects of melatonin, combined with its anti-oxidant, NMDA-blocking and anti-inflammatory properties, probably make this molecule an ideal candidate for pre-clinical studies. Moreover, this pharmacological agent has been proven to be safe for neonates when crossing the blood-brain barrier. Several experimental data have shown potent neuroprotective effects of melatonin both in *in vivo* and *in vitro*. In particular, melatonin acts as a direct and indirect antioxidant, specifically as a powerful scavenger of superoxide anion and stimulator of the synthesis of anti-oxidant enzymes. However, the consequences of melatonin treatment on the myelination process remain largely underexplored. Nonetheless, this question is crucial to thoroughly assess the potential impact of melatonin on myelination repair in perinatal and adult demyelinating diseases.

We addressed this question by testing melatonin treatment in a recently described rat model closely mimicking WMD observed in very preterm neonates [8]. Our data show that melatonin has a powerful protective effect on developing white matter subjected to an antenatal hypoxic-ischemic insult. Melatonin acts through its specific receptors by promoting oligodendroglial maturation and myelination repair together with modulation of astrogliosis and microglial activation *in vivo* and *in vitro*. Our results suggest that melatonin could be an effective neuroprotective candidate not only in perinatal white matter damage but also in inflammatory and demyelinating white matter diseases observed in adult.

## Materials and Methods

### Uterine artery ligation

Unilateral ligation of the intra-uterine artery was performed on pregnant Sprague-Dawley rat (Janvier, Le Genest-St-Isle, France) during embryonic day 17 (E17) [8]. The mean weight of control pups at birth in our animal facilities was 6.24±0.37 g (n = 56). In each litter, the pups were separated into two groups according to birth weight: control pups and severely growth restricted (GR) pups weighting less than 5.50 g (6.34 g–2 S.D.). Only the most severely GR pups were considered as born from the ligated horn to avoid any bias between the two groups. All experiments were carried out in compliance with ethical standards of our institution (French National Institute for Health and Medical Research, INSERM) and with the recommendations of the National Research Council's Guide for the Care and Use of Laboratory Animals.

### Experimental groups

Pups from at least three different litters were used in each experimental group. From birth (P0) to postnatal day 3 (P3), controls and GR pups received a daily i.p. injection with one of the following drugs (or combination of drugs) diluted in a final volume of 5 µl: 0.002 mg/kg/d to 20 mg/kg/d melatonin (Sigma), 5.0 mg/kg luzindole (Sigma) +/− 20 mg/kg/d melatonin. Luzindole blocks melatonin receptors MT_1_ and MT_2_ with comparable affinities (affinity selectivity ratio MT_1_/MT_2_ = 15.5). To ensure that there were no differences in circadian rhythm between experimental groups, all rat pups were injected once a day at noon.

### Immunocytochemistry

In each experimental group, we studied 6 to 10 pups on P3 and P14 in three separate experiments. Animals were sacrificed under anaesthesia with inhaled isoflurance (Abbott France, Rungis, France). Brain tissues were immersed in formol 4% during 5 days, embedded in paraffin and cut into 10-µm-thick sections.

Immunolabeling with the primary antibody listed in Table 1 was visualized using the streptavidin-biotin-peroxydase method, as previously described [8]. Olig2 marker was used to visualize all oligodendrocytes, APC and MBP to detect post-mitotic oligodendrocytes and myelinated fibers, respectively. Most of Olig2 nuclei did not colocalized with GFAP+ cells (Figure S1). Tomato-lectin-, Olig2- and Ki67-immunoreactive cells were revealed using streptatividin, anti-rabbit IgG antibody and anti-mouse IgG antibody coupled to the red fluorescent marker cyanine 3 (Jackson Immunoresearch laboratories, West Grove, PA). Double labelings were performed with secondary antibodies coupled to the green fluorescent marker Fluoroprobe S488 (Interchim, Montluçon, France) or red fluorescent marker cyanine 3 (Jackson Immunoresearch laboratories, West Grove, PA) fluorescent markers.

**Table 1:** Primary antibodies used for immunohistochemistry analyses.

Markers	Labeled structures	Manufacturer	Dilution
Tomato-lectin	Resident, activated microglia and blood vessels.	Vector, Burlingame, CA, USA	1/500
GFAP	Astrocytes	Sigma Biosciences, St Louis, MO, USA	1/1000
Ki67	Proliferating cells	BD Biosciences France, Le-Pont-De-Claix, France.	1/100
Olig2	oligodendroglial lineage	IBL, Hamburg, Germany	1/200
APC	post-mitotic oligodendrocytes	Calbiochem, La Jolla, CA, USA	1/500
MBP	Myelinated fibers	Sigma Biosciences, St Louis, MOUSA	1/1000
MT1 (Mel-1A)	Melatonin receptor 1	Santa Cruz, Santa Cruz, CA, USA	1/100
MT2 (Mel-1B)	Melatonin receptor 2	Santa Cruz, Santa Cruz, CA, USA	1/100

### Quantitative measurements

All quantitative measurements were done by observers who were blinded to the experimental group of the studied animal.

#### Immunoreactive cells

Immunoreactive cells were counted in the white matter underlying the cingular cortex (+2.16 to −0.36 mm from the bregma) on at least 4 non continuous sections (alternatively, in right or left hemisphere) at P3 and P14. Immunoreactive cells were counted within a 0.065 mm^2^ grid (at ×400 magnification), in at least 6 animals per group.

#### Optical density of MBP-positive fibers

The optical density of MBP-immunoreactive fibers was measured in the thickest white matter in rodents, ie cingular white matter assessed in coronal sections (+2.16 to −0.36 mm from the bregma). At least 4 sections of 6 to 10 animals per group were examined on P14. Optical density was measured at ×100 magnification using a computerized image analysis system (ImageJ, NIH, MA, http://rsb.info.nih.gov/ij/) that read optical density as grey levels. Nonspecific background densities were measured at each brain level in a region devoid of MBP immunostaining and were substracted from the cingulum values.

### TUNEL staining

In P3 animals, dying cells in the white matter were detected using TUNEL as previously described [3] and counted in 4 sections each from a single hemisphere of at least 6 animals per treatment group.

### Ultrastructural morphology of cingulate white matter by electron microscopy

The morphology of melatonin-treated cingulate white matter was studied by electron microscopy. First, brain were sagitally cut in 100 µm-thick vibratome sections to allow for better penetration of the reagent and postfixed for 10 min at room temperature with 1% osmium tetroxide (Heraeus Chemicals, Port Elisabeth, South Africa) in phosphate buffer. After a 10 min wash in a phosphate buffer, sections were dehydrated in graded alcohol baths and successively incubated in alcohol/araldite (v/v) for 1 h at 37°C, araldite (Fluka, Steinheim, Switzerland) for 15 h at 37°C, and araldite supplemented with 2% accelerator (Fluka) for 3 h at 37°C. Sections were then embedded in araldite (2 days at 60 °C). Silver ultrathin sections of the cingulate white matter (about 70 nm-thick) were made using an ultramicrotome (LKB, Bromma, Sweden) and then collected on copper grids. They were counterstained with lead citrate for 10 min at room temperature in a drying chamber and examined using a Leo 912 (Carl Zeiss, Le pecq, France) electron microscope. In order to study the qualitative aspect of the myelin, the number of axons surrounded with an uncompacted myelin sheet was reported as the total number of myelinated axons counted of 20 electromicrographes at magnification ×4000. A qualitative score was applied to each myelinated axon (see table 2).

**Table 2:** Quantitative analysis of the optical density of myelin sheets in the cingulate white matter of controls and GR pups with or without melatonin 20 mg/kg.

Group	Myelin sheet O.D.
Controls	95.2±0.4
GR pups	58.7±0.7*
GR pups+melatonin	82.5±0.8

Data are expressed mean ± S.E.M. Asterisk represent significant difference with controls (* p<0.05 with one way ANOVA and Newman-Keuls multiple comparision test).

### Cell cultures and cell treatments

Primary neuronal cultures (>98% purity assessed by MAP-2 (microtubule-associated protein-2) immunostaining, data not shown) were prepared using E18 embryonic rats. For oligodendrocyte, astrocyte and microglia primary cultures, cell populations were isolated from P0–P2 newborn rats and subsequently cultured according to published protocols from 6 days to 3 weeks [9]–[11]. Purity of oligodendroglial cell cultures was assessed using O4, GFAP and tomato-lectin staining: at DIV10, 65–70% of the cells were found O4+, 25–30% GFAP+ and less than 5% Tomato-lectin+ in three separate experiments.

DNA-free total mRNA from cell cultures and cortical plate from rat aged from 1, 7 and 14 day-old rats were extracted from these cells using the previously published protocol [12].

Oligodendoglial cells were exposed to melatonin at 1 and 100 µmol diluted in DMSO 20% after 6, 8 and 10 days of culture. As our in vivo model is characterized by early microglial activation within developing white matter we further tested the ability of melatonin to modulate microglial activation *in vitro*. We used a well-established model of microglia subjected to LPS leading to both morphological and immunocytochemical changes and a dramatic increase in cytokines and free radicals production. In microglial cell cultures, the melatonin treatment was performed after 5 days of culture, 12 h before and at the same time as LPS exposure (100 ng/ml LPS) (Sigma, St Louis, MO).

### Immunocytochemistry and immunofluorescence microscopy on cell cultures

After treatments, cells were fixed with 4% paraformaldehyde in PBS for 10 min at room temperature, washed 3 times with PBS, and blocked with PBST (PBS 1x, pH 7.4, 0.1% Triton X-100) containing 5% goat serum for 1 hr at room temperature. The coverslips were incubated with mouse monoclonal antibodies O_4_ (1∶100 dilution, gift from Dr. Steven E. Pfeiffer, University of Connecticut Health Center, Farmington, CT), tomato lectin (1∶1000, Vector, Burlingame) and MBP (1∶1000, Chemicon, Temicula, CA) overnight at 4°C. The appropriate secondary antibody was conjugated with either Alexia 488 or Alexia 594 (Molecular Probes Inc., Eugene, OR), added to the coverslips and incubated for 1 hr. Nuclei were stained by adding Hoechst 33258 at a final concentration of 2 µg/ml for 1 min. The coverslips were mounted onto glass slides with FluoroMount and kept dark at 4°C. Cell images were captured with a fluorescence microscope equipped with a digital camera (Leica DMRB with Apogee Instruments Inc.). Quantification of labelled cells were based either on the density of immunoreactive cells compared to the total number of nuclei (O4 and MBP, ×400 magnification) or the optical density of tomato-lectin immunoreactivity using ImageJ analysis system at ×200 magnification.

### Quantitative real-time PCR

The DNA-free total RNA from control and GR brain cortex including white matter was obtained using a protocol adapted from Chomczynski and Sacchi^12^. The following oligonucleotides 5′-ATTGTCAAGTTAGTGCCTTCC-3′ and 5′-TTGAGACTGTGGCAAATGTAG-3′, 5′-GTCATTGGCTCTGTCTTCAAC-3′ and 5′-GTAGGTCGCACTGTGACAGAT-3′ were used as sense and antisense primers, for MT1 and MT2. The nature of the amplified DNA was confirmed by sequencing. To standardize gene expression across samples, we first compared the expression levels of four well-known housekeeping genes (glyceraldehyde-3-phosphate dehydrogenase [GAPDH], β2-microglobulin, hypoxanthine-guanine phosphoribosyltransferase [HPRT], and β-glucoronidase) within the samples. For reverse transcription, we used 600 ng of total RNA and the Iscript cDNA synthesis kit (Bio-Rad, Hercules, CA). Real-time PCR was set up using sybergreen-containing supermix (Bio-Rad) for 50 cycles with a three-step program (25 sec denaturation at 96°C, 30 sec annealing at 60°C, and 30 sec extension at 72°C). Amplification specificity was assessed by melting curve analyses. Each experiment was run twice with a least 6 animals per group, and in both cases, samples were assessed in triplicate.

### Statistical analysis

All data were reported as means ± S.E.M. Analysis of variance was performed with age and groups (PBS- and melatonin-treated controls and GR pups) as the factors, and the Newman-Keuls post-test was used. Statistical tests were run on GraphPad Prism version 4.00 (GraphPad Software, San Diego, CA).

## Results

### Melatonin attenuates myelination defect in growth restricted pups

**Figure 1:**
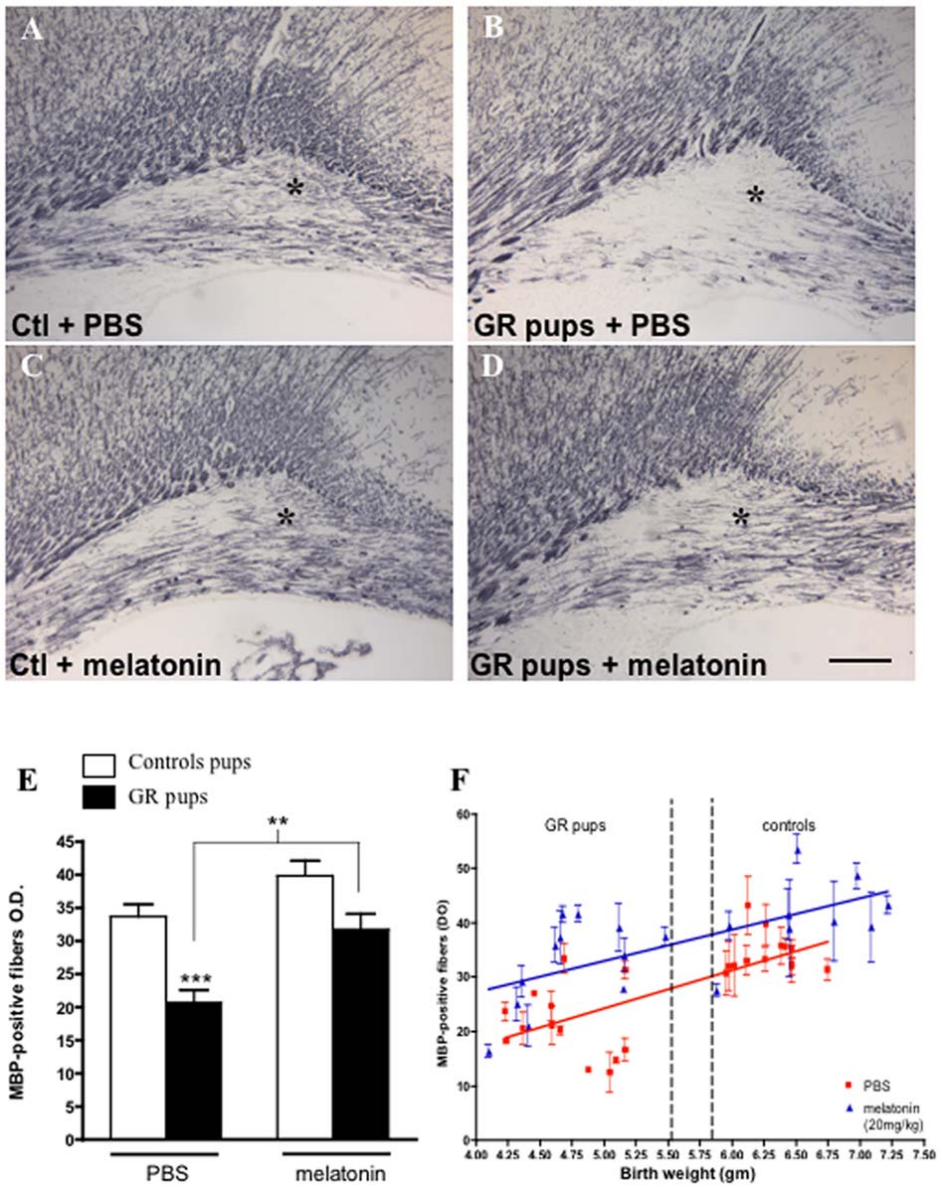
Melatonin attenuates myelination defect in the cingulum. (A–D) Cingulate white matter MBP-immunoreactivity in P14 rat pups demonstrating that myelin content was defective in PBS-treated GR pups (B) compared to PBS- (A) and melatonin-treated controls (C). In contrast, melatonin-treated animals (D) showed an MBP-immunoreactivity density similar to controls. Bars = 200 µm. (E) Quantitative analysis of the MBP-positive fibers optical density in the cingulate white matter of controls and GR pups treated with PBS or with melatonin 20 mg/kg. The area of optic density measurement is indicated by an asterisk in (A–D). Statistical analysis compared either control and GR pups or untreated and treated GR pups. (**p<0.01 and ***p<0.001 using one-way ANOVA with the Newman-Keuls correction). (F) Scattered plot representation of MBP-positive fibers immunoreactivity density in the cingulate white matter of PBS- and melatonin-treated controls and GR pups at P14 according to their birth weight. Squares (in red) and line represent PBS-treated animals, triangles (in blue) and line represent melatonin-treated animals. Dotted-lines represents weight of 5.50 g and 5.87 g, respectively the upper limit (−2 SD) of GR animals birth weight and the lower limit (−1 SD) of control animals birth weight.

**Figure 2:**
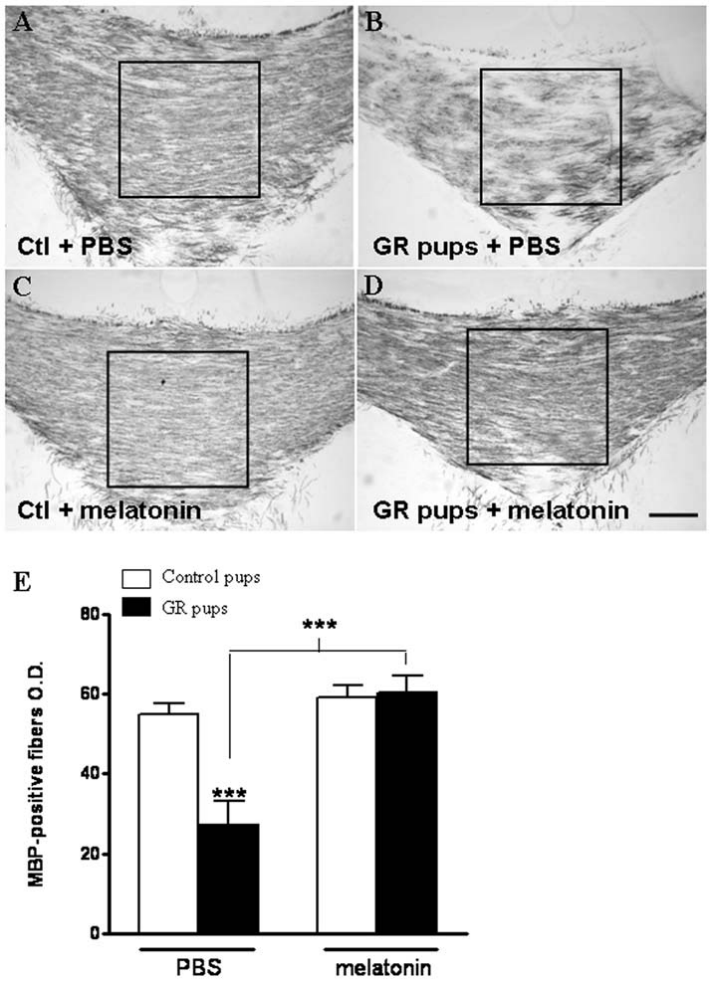
Melatonin attenuates myelination defect in the corpus callosum. (A–D) White matter MBP-immunoreactivity in P14 rat pups demonstrating that myelin content was defective in the genu of the corpus callosum in PBS-treated GR pups (B) compared to PBS- (A) and melatonin-treated controls (C). In contrast, melatonin-treated animals (D) showed an MBP-immunoreactivity density similar to controls. Bars = 200 µm. (E) Quantitative analysis of the MBP-positive fibers optical density in the corpus callosum white matter of controls and GR pups treated with PBS or with melatonin 20 mg/kg. The area of optic density measurement is indicated by a square in (A–D). Statistical analysis compared either control and GR pups or untreated and treated GR pups. (***p<0.001 using one-way ANOVA with the Newman-Keuls correction).

**Figure 3:**
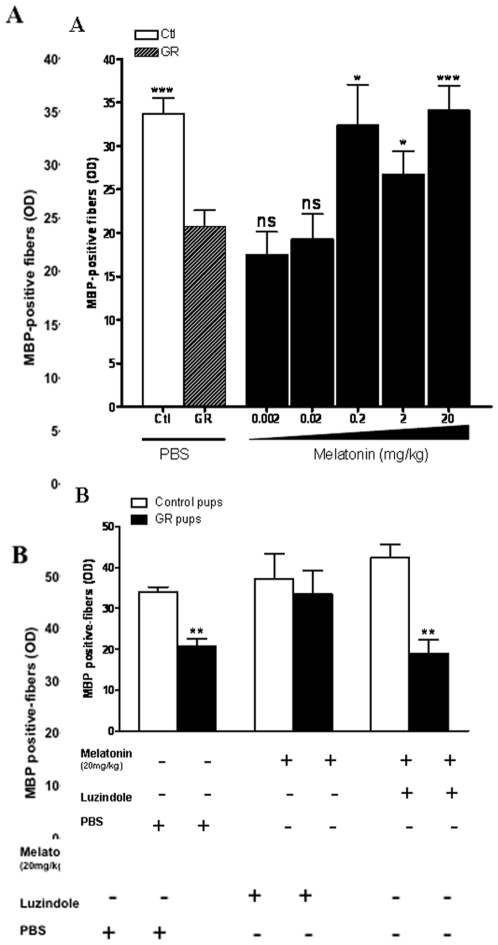
Melatonin effect is dose-dependent and mediated by its specific receptors. (A) Cingulate white matter MBP-immunoreactivity optical density in P14 GR pups compared to controls demonstrating that melatonin was able to restore normal MBP content with a minimal dose of 0.2 mg/kg. Controls and treated GR animals were compared to untreated GR pups. *p<0.05 and ***p<0.001 using one-way ANOVA with the Newman-Keuls correction. (B) The melatonin effect on myelination was reversed when melatonin was co-injected with luzindole, a specific MT1/MT2 antagonist. **p<0.01, NS: not significant using one-way ANOVA with the Newman-Keuls correction.

Systemic treatment using 20 mg/kg/d melatonin given within the first 3 postnatal days was associated with a significant 58% attenuation of myelination defects detected in the cingulate white matter in P14 GR pups (p<0.001) (Figures 1A–E). Similar results were observed in the genu of the corpus callosum (Figure 2A–E). This effect was observed in most of GR pups, however, the most severely growth restricted (below 4.5 g, corresponding to birth weight below 5 SD) only showed a minor benefit from the melatonin treatment (Figure 1F). For birth weights greater than 4.5 g, the melatonin treatment was associated with a remarkably stabilized MBP density regardless the birth weight, in contrast to untreated animals in which myelin content appeared closely correlated with birth weight (Figure 1F). Administration of melatonin induced a dose-dependant effect on the density of MBP-positive fibers in the cingulate white matter in GR pups (Figure 3A). A statistically significant effect of melatonin was observed with a minimal administration of 0.2 mg/kg/d melatonin. No effect of melatonin on both body temperature and weight gain during the first 7 days has been reported (data not shown). Similarly, brain weight gain was not found different during the first 14 days with or without melatonin treatment (table S1). No statistically significant difference in oligodendroglial cell death (TUNEL staining) was observed between treated and control rat pups at P3 (Figure S2). Finally, melatonin treatment during the first days of life (P0–P3) was not associated with any alteration in myelin content in the normal developing brain (both in cingulum and corpus callosum, Figure S3).

In melatonin-exposed GR rats, the blockage of melatonin receptors, using luzindole co-treatment, eliminated the neuroprotective effect of melatonin on white matter myelination (Figure 3B). In the control rat pups, melatonin alone, luzindole alone or co-treatment did not modify MBP-immunoreactivity cingulate white matter. Luzindole alone did not potentiate the myelin defect observed in GR pups (data not shown).

### Melatonin improves the ultrastructural myelin aspect in GR pups

**Figure 4:**
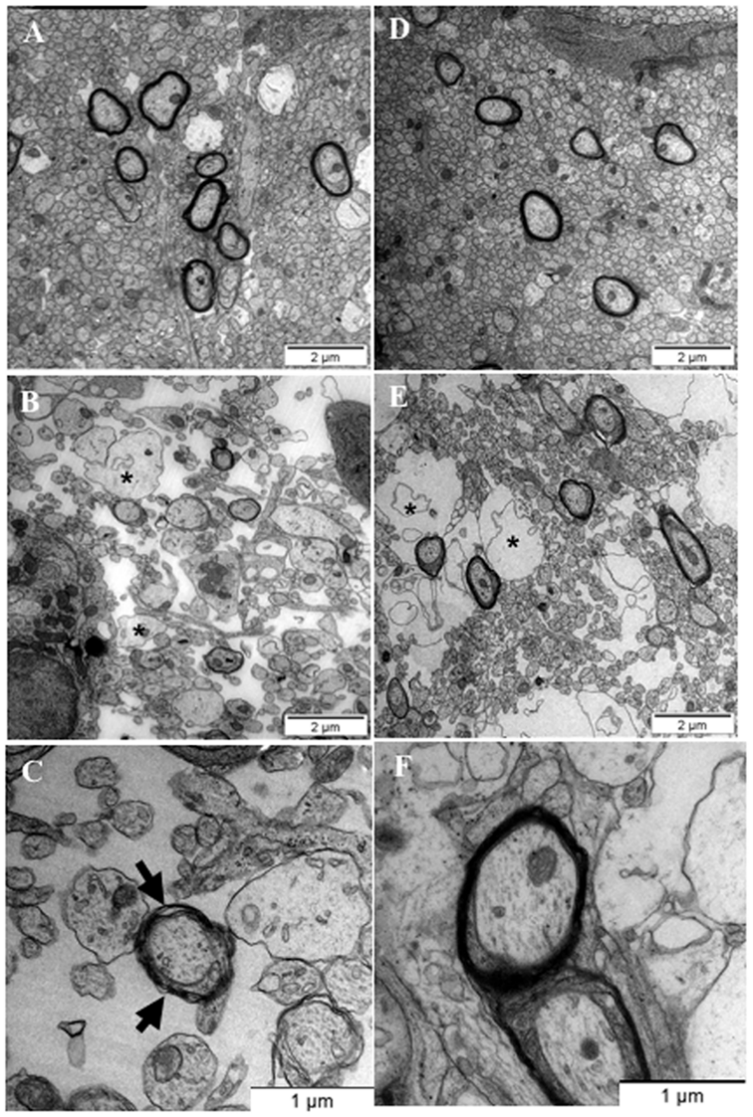
Melatonin improves the ultrastructural myelin aspect in GR pups. (A–F) Cingulate white matter ultrastructural aspect observed using electron microscopy. PBS-treated GR pups (B, C) exhibited swollen axons (marked with an asterisk) and dramatically low cellular, axonal and myelinated axons density compared to PBS- (A) and melatonin-treated controls (D). Note the large proportion of myelinated axons surrounded with uncompacted myelin sheets (arrows) in PBS-treated GR-pups (B, C). In contrast, melatonin-treated GR animals exhibited a high proportion of well-compacted myelination processes, similar to controls (E–F). Interestingly, melatonin treatment appeared to not be able to restore a normal axonal aspect within the developing white matter (E). Swollen axons were marked with an asterisk. Quantitative analysis of myelin ultrastructural aspect was reported in table 2.

Further experiments using electronic microscopy demonstrated ultrastructural quantitative and qualitative effects of melatonin on myelinated axons of rat pups that are subjected to antenatal uterine ligation. In PBS- or melatonin-treated control P14 pups, the developing white matter exhibited a high density of cells and myelinated ovoid axons with well-compacted myelin sheets (Figures 4A, 4D). In contrast, PBS-treated GR pups showed dramatic cingulate white matter damage including a disorganized structure of the developing white matter with undermyelinated and swollen axons (Figure 4B). In most of cases, myelinated axons were surrounded with uncompacted myelin sheets (table 2). Melatonin treatment of GR pups was associated with a quantitative and qualitative improvement of the myelin ultrastructural aspect. Indeed, myelin features in treated animals were characterized by a good compaction of the myelin sheet around axons (table 3; Figures 4C, 4F). Thirty two percent of myelinated axons observed in untreated GR animals were found embedded with compacted myelin sheets compared to 79% in controls and 69% in treated GR animals. Conversely, melatonin treatment was unable to restore cellular and axonal density suggesting a specific effect on myelination process (Figures 4E, 4F). Similarly, melatonin treatment was not able to change the immunocytochemical aspect of RT-97-positive axons in both control and GR pups (data not shown).

**Table 3:** Ultrastructural analysis of myelinated fibers in controls and GR pups with electron microscopy.

Group	Qualitative myelin aspect score	% of myelinated axons embedded with compacted myelin sheets
Controls	2.6±0.1	79.1(±7.5)%
GR pups	1.1±0.5*	32.2(±7.8)%*
GR pups+melatonin	2.6±0.1	68.6(±12.2)%

Qualitative myelin aspect score:

0: no myelinated axons found;

1: very thin myelin sheet;

2: uncompacted myelin sheets;

3: normally compacted myelin sheets.

Data are expressed mean ± S.E.M. Asterisk represent significant difference with controls (* p<0.05 with one way ANOVA and Newman-Keuls multiple comparision test).

### Melatonin treatment promotes oligodendroglial maturation in GR pups

**Figure 5:**
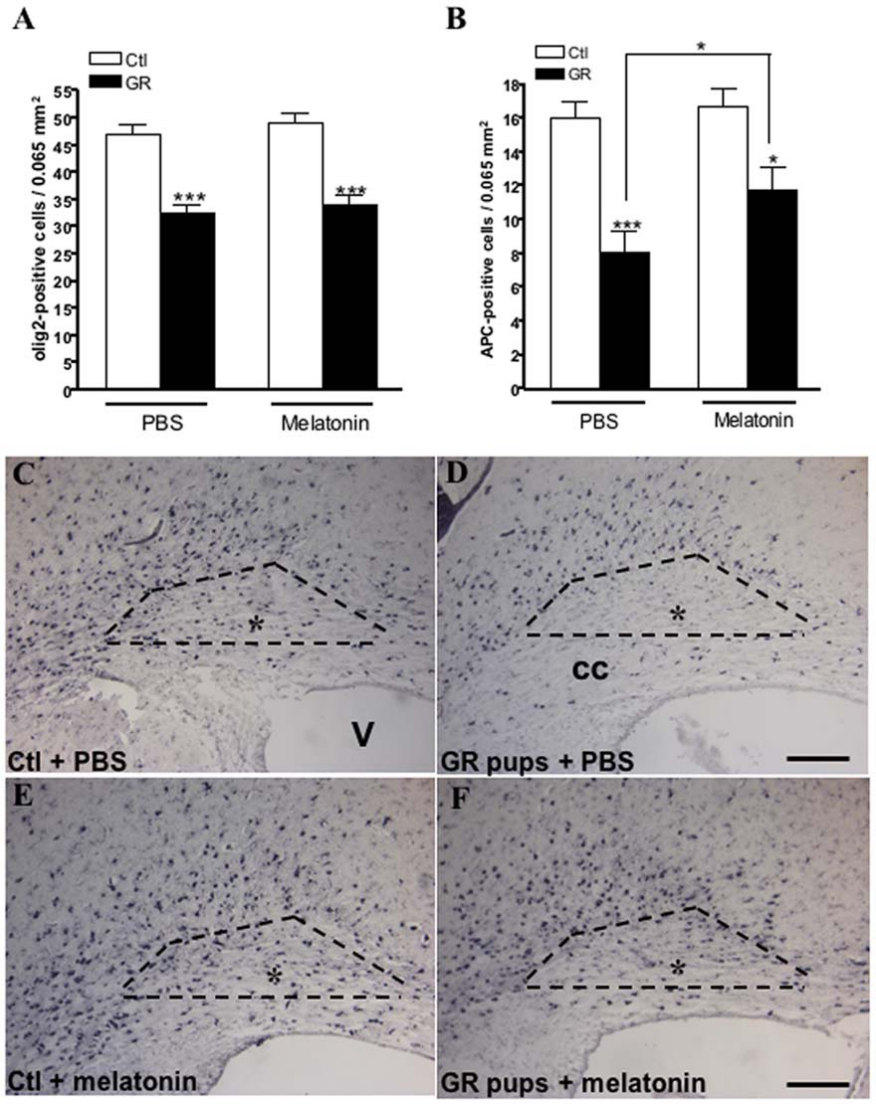
Melatonin promotes oligodendroglial maturation. (A) Quantitative analysis of Olig2-immunoreactive cells in the cingulate white matter at P14. Melatonin treatment was found to not be able to reverse the loss of total oligodendrocytes observed in GR pups. (B) Quantitative analysis of APC-immunoreactive cells in the cingulate white matter at P14. Melatonin treatment was associated with a significant reduction in mature oligodendrocytes deficit observed in GR pups. *p<0.05 and ***p<0.001 using one-way ANOVA with the Newman-Keuls correction. (C–F) PBS-treated GR pups exhibited a defective mature (APC-immunoreative) oligodendrocyte density (D) compared to PBS- and melatonin-treated controls (C, E). Melatonin treatment partially restored mature oligodendrocytes density (F). Microphotographes show cingulate white matter with the standardized counting area indicated by dotted lines. V: ventricule. cc = corpus callosum underlying the cingulum. Bars = 200 µm.

To further delineate the cellular target of melatonin treatment, we asked the question whether melatonin could impact the oligodendroglial lineage. To answer this question, the effect of melatonin treatment on oligodendrocyte density was assessed using various immunocytochemical markers (see methods). The cingulate white matter of GR pups appeared to be depleted in total (Olig2+) and mature (APC+) oligodendrocytes at P14 (Figures 5A, 5B) as well as P3 (data not shown). Melatonin treatment was not able to restore the Olig2 positive-oligodendrocyte density in the cingulate white matter as well as the adjacent corpus callosum in GR pups (Figure 5A). In contrast, melatonin-treatment significantly (p<0.05) reduced the decrease of APC+ oligodendrocytes density observed in cingulate white matter of GR rat pups at P14 (Figures 5B–F). Moreover, using Olig2/APC double-labelling immunocytochemistry, 63% of Olig2+ oligodendrocytes were found APC+ in untreated GR pups compared to more than 92% in melatonin treated P14 GR pups (p<0.05). Conversely, melatonin was not found to be associated with any change in cell proliferation in the developing white matter as assessed using Ki67 immunolabeling at P3 and P14 (Figure S4). Altogether, these data strongly suggest that melatonin treatment promotes oligodendroglial maturation in GR rat pups.

### Melatonin treatment is associated with a decrease of glial activation in GR pups

**Figure 6:**
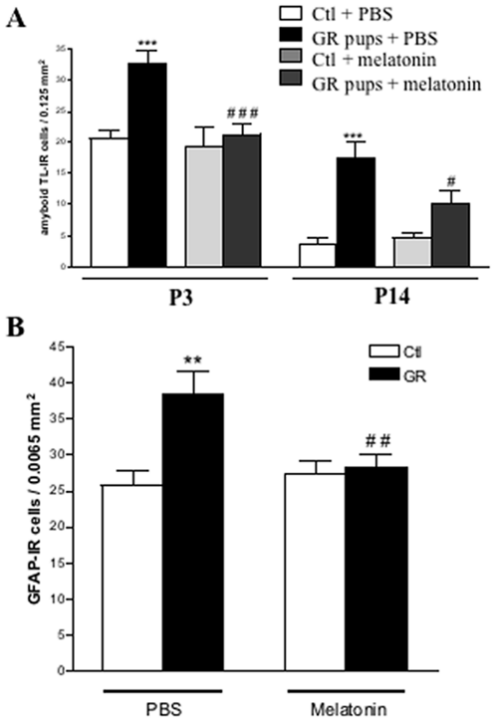
Melatonin is associated with a decreased glial activation. (A) Quantitative analysis of amyboid tomato-lectin-immunoreactive cells in the cingulate white matter in P3 and P14 rat pups. PBS-treated GR pups exhibited a significant increase in density of reactive microglia compared to PBS- and melatonin-treated controls. In contrast, microglial activation in melatonin-treated GR animals was similar to controls. (B) Quantitative analysis of GFAP-immunoreactive cells in the cingulate white matter in P14 rat pups. PBS-treated GR pups exhibited a significant increase in reactive astrocyte density compared to controls. Melatonin-treatment was associated with a normal astroglial density in treated-GR pups compared to controls. Analysis was performed using two-way ANOVA with the Newman-Keuls correction *p<0.05, **p<0.01 and ***p<0.001 indicating significant differences between GR and control rat pups. #p<0.05, ##p<0.01 and ###p<0.001 indicating significant differences between PBS- and melatonin-treated GR animals.

Growth restriction induced by antenatal hypoxia was associated with a protracted increase of microglial activation in the cingulate white matter [7]. Thus, we investigated the consequences of melatonin treatment on microglial activation in antenatal hypoxia-exposed neonatal rats. In cingulum white matter, melatonin-treated GR pups exhibited a significantly decreased density of activated microglial cells compared to PBS-treated GR rat at P3 and P14 (Figure 6A). During the same developmental stages, melatonin treatment did not modify microglial activity in the cingulate white matter of controls. An increased density of GFAP-positive astrocytes in the cingulate white matter was observed in P14 GR rats compared to controls. Furthermore, reactive astrocytes from GR pups had a protoplasmic profile, with a bigger cell body and more numerous processes compared to control pups. At similar developmental stages, melatonin treatment was associated with a significantly decrease in reactive astrocyte density in GR pups compared to controls (p<0.01) (Figure 6B). Melatonin-treated controls did not exhibit any modification of cingulate white matter astroglial profile and density.

### Melatonin receptors are differentially expressed in the white matter glial cells

**Figure 7:**
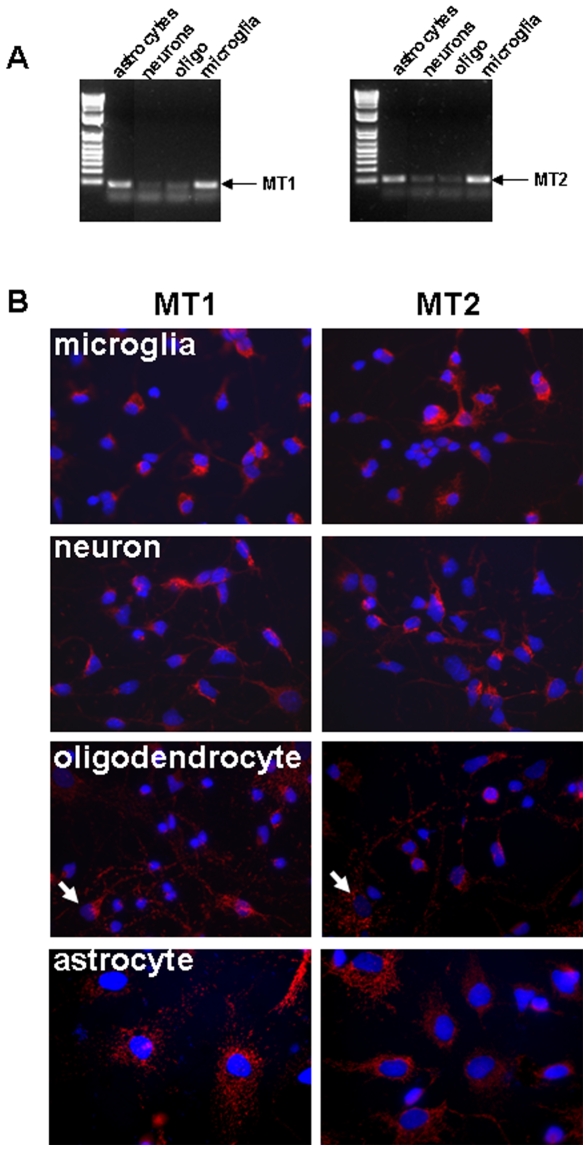
Melatonin receptors are differentially expressed in white matter glial cells *in vitro*. (A) Relative expression of MT1 (amplicon: 109 bp) and MT2 (amplicon: 75 bp) mRNA in various cell cultures including astrocytes, neurons, immature oligodendrocytes and microglia.(B) Immuno-labelling of various primary cell cultures (microglia, neuron, oligodendrocytes, astrocytes) using either MT1 or MT2 markers. Arrows show small fraction of astrocytes contaminating oligodendrocyte cell cultures.

Because the effect of melatonin is recognized to be mediated through specific G-protein coupled receptors MT1 and MT2 (antagonized by luzindole), we next asked the question whether these two receptors were expressed in the various cell types populating the developing white matter at the time melatonin treatment was given. We tested this using RT-PCR *in vitro* in neurons, microglia, astrocytes and immature oligodendrocytes, all cell types populating the developing white matter. MT1 and MT2 appeared to be strongly expressed in both astrocytes and microglial cells, and to a lesser extent in neurons and immature oligodendrocytes (Figure 7A). Immunocytofluorescent stainings using MT1 (Mel-1A) and MT2 (Mel-1B) antibodies revealed similar expression pattern (Figure 7B).

### Melatonin induces oligodendroglial maturation and modulates microglial activation *in vitro*

**Figure 8:**
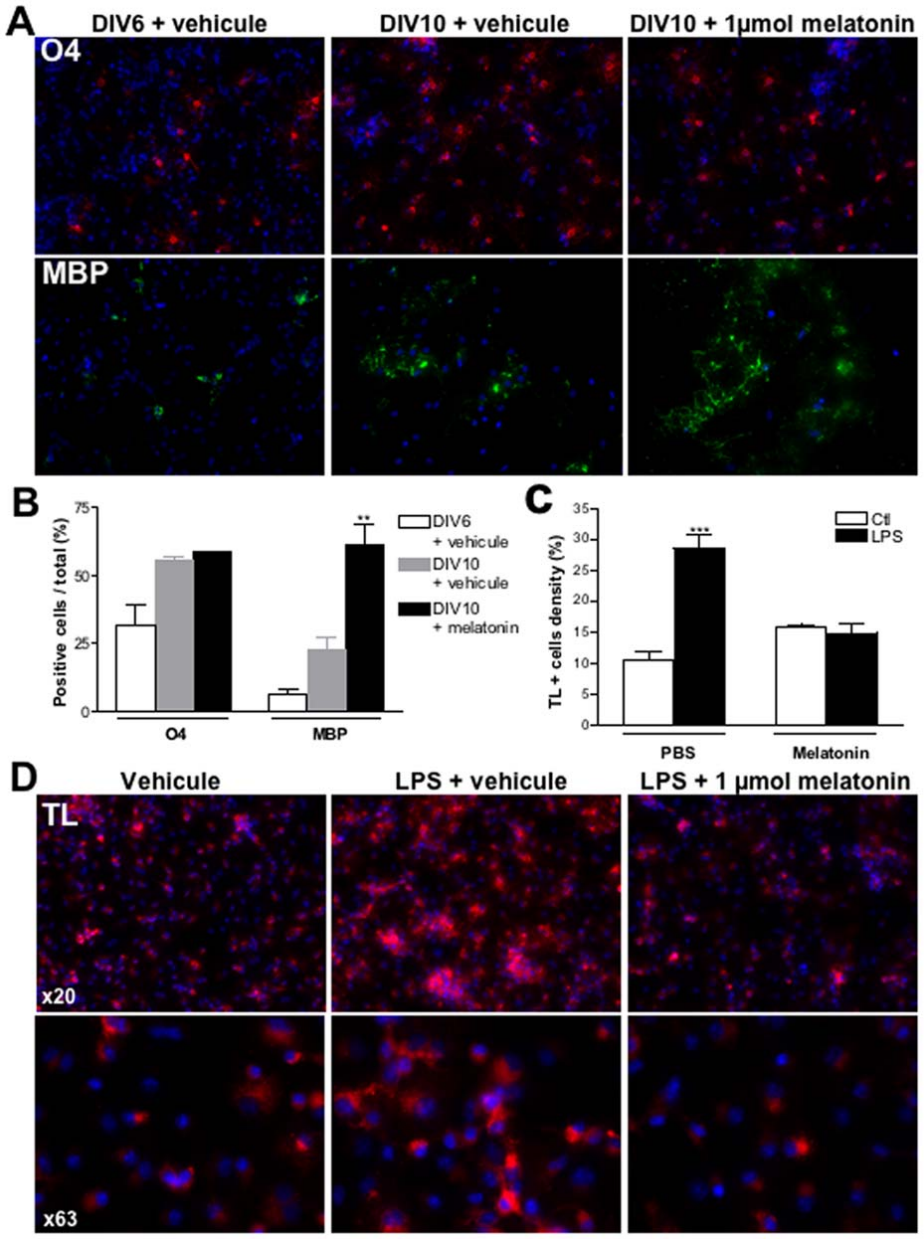
Melatonin promotes myelin production and prevents LPS-induced microglial activation *in vitro*. (A) Immuno-labelling of primary oligodendroglial cell cultures using either O4 or MBP at DIV6 or DIV10 with or without treatment with 1 µmol melatonin. (B) Quantitative analysis of the density of O4 or MBP immunoreactive cells at DIV6 and DIV10 with or without treatment with 1 µmol melatonin. **p<0.01, using one-way ANOVA with the Newman-Keuls correction. (D) Immuno-labelling of microglial cell cultures using tomato-lectin in presence of LPS with and without 1 µmol melatonin. Note the marked increase in tomato-lectin immunoreactivity in microglia subjected to LPS. (C) Quantitative analysis of the density of tomato-lectin positive cells in presence of LPS with and without 1 µmol melatonin. ***p<0.001, using two-way ANOVA with the Newman-Keuls correction.

Developing oligodendrocytes and microglia were the two major cell types involved in the main features of white matter damage in our rat model. Since we found melatonin receptors to be expressed in both cell types, we further investigated the impact of melatonin exposure *in vitro*. In the oligodendroglial lineage model we observed that 1 µmol melatonin exposure was associated with an enhancement of oligodendroglial maturation to myelinating oligodendrocytes (Figures 8A, 8B). Indeed, MBP positive cell density increased more than two-fold at DIV 10 after melatonin treatment compared to DMSO or untreated cells (p<0.01). In addition, APC and PLP immunostainings were also found increased after melatonin treatment in oligodendrocyte cell cultures (Figure S5). Similar results were obtained using 100 µmol melatonin (data not shown).

Microglial activation was considered as a key feature in our *in vivo* model, likely to be responsible for subsequent myelination deficit. To mimic this feature *in vitro*, we used LPS to activate microglial cells. Because melatonin treatment was associated with a dramatic effect on microglial activation *in vivo*, we tested the ability of melatonin to modulate the effect of LPS-induced microglial activation *in vitro*. Microglial cells were pre-treated with either 1 or 100 µmol melatonin 12 h hours before and then together with LPS exposure. LPS-treated microglial cells exhibited an amyboid shape and a significant increase in tomatolecin immunoreactivity density compared to non-activated cells. In contrast, melatonin exposure completely reversed the LPS effect and prevented activation of microglial cells (Figures 8C, 8D). A similar effect was observed using two different cellular densities of microglial cells (data not shown).

## Discussion

In this study we investigated the underlining cellular mechanisms responsible for the neuroprotective effects of melatonin treatment in antenatal hypoxia-induced white matter injury in the developing rat brain. We have demonstrated that melatonin acts in a neuroprotective fashion by promoting oligodendroglial maturation together with decreased microglial activation, both *in vivo* and *in vitro*. We recently reported a rat model inducing IUGR and white matter damage after prenatal hypoxia and deprivation of nutritional and maternal factors [13]. As previously described, GR pups exhibited white matter damage characterized by increased microglial activation, increased astrogliosis, leading to a protracted oligodendrocytes depletion associated with myelination defects [7], [14]. In this study, electron microscopy analysis of the injured white matter refined the abnormal aspect of the developing white matter. The few myelinated axons were mostly embedded with a poorly compacted myelin. MBP proteins have been recognized to have a crucial role in myelin compaction by intercalating between phospholipidic sheets and interacting with lipids and proteolipids [15], [16]. Similarly, Shiverer mice, a natural mutant for MBP gene, exhibit hypomyelination and produce poorly compacted myelin around axons [17], [18].

In GR animals, the white matter also appeared with large areas of swollen axons, suggesting protracted oedematous damage. Despite no detectable change in axonal neurofilament distribution, ultrastructural analysis of neonatal white matter clearly demonstrates that growth restriction observed in our model is associated with oligopathy and myelinopathy.

Several experimental studies have highlighted that melatonin has neuroprotective benefits when given as either a prophylactic or curative treatment in several animal models of brain damage. Most of such studies have analyzed the effect of melatonin in traumatic or brain focal ischemia in adult rodent [19]–[21]. In such models, melatonin was found to decrease the volume of brain damage, neuronal cell death, oxidative stress, DNA damage, mitochondrial insult and to improve neurobehavioural outcome. Furthermore melatonin exerts its neuroprotective benefits against excitotoxic insult both *in vitro* [22], [23], *ex vivo* in hippocampal neurons in adult rats [24] or *in vivo* [4]. Melatonin has also been studied in a large animal model of preterm brain injury in fetal sheep subjected to umbilical cord occlusion [25]. This study demonstrated that melatonin attenuates cell death in the fetal brain in association with a reduced inflammatory response in the blood and the brain following intrauterine asphyxia in mid-gestation fetal sheep.

In mature melatonin-deficient rats, lesions induced by hypoxia-ischemia were observed to be larger than in controls [26] suggesting that endogenous melatonin is neuroprotective. However newborn animals and humans have low levels of melatonin and the melatonin receptor antagonist luzindole does not potentiate excitotoxic cerebral white matter lesions in rodents, suggesting that endogenous melatonin is not sufficient to protect the newborn. For exactly this reason, melatonin treatment could be relevant in a clinical setting in preterm human neonates.

Melatonin has been shown to have no effect in preventing the initial appearance of lesions in a mouse model of excitotoxic-induced white matter damage, but is able to promote axonal regrowth or sprouting [4]. However, the effect of melatonin on myelination and subsequent white matter repair has not yet been studied. Previous studies have shown that white matter damage observed in our model was characterized by a depletion in post-mitotic oligodendrocytes (APC+ cells) rather than pre-oligodendrocytes (O4+ cells) [14]. In the present study, the olig2 immunolabeling suggest that the entire oligodendroglial lineage is defective in GR animals' white matter. It is relevant to point out that melatonin-induced preservation of myelination is associated with partial recovering of mature APC+ oligodendrocytes (despite a deficit in total oligodendroglial population). Consequently, we speculate that the main target of melatonin treatment could be the maturation process of the oligodendrocyte lineage. This hypothesis is supported by several lines of evidence: melatonin treatment increased oligodendrocyte maturation and MBP-immunoreactivity normalization *in vivo*. Melatonin treatment was associated with a restoration of myelin sheet compaction surrounding axons in GR pups white matter. This result is consistent with MBP-immunoreactivity normalization as MBP is crucial for myelin sheet compaction [15], [16]. Finally, melatonin was able to improve oligodendrocytes maturation without any effect on cell proliferation *in vitro* or *in vivo*.

The mechanism(s) behind the neuroprotective benefits of melatonin are not yet fully elucidated. To date, it has been shown that the effect of melatonin include endocrine, autocrine and paracrine actions, decreased inflammation, and antioxidant effects. Melatonin has also some benefits in other “toxic models” of perinatal brain damage based on excitotoxic cascade [4]. However, in most of perinatal brain damage animal models reported, immune cell activation frequently occurs [1] and melatonin effect appears to be closely linked to inflammation modulation. Some of these actions are receptor-mediated, while others are direct. We demonstrate here that the two major subtypes of melatonin-receptors MT1 and MT2 were expressed by microglia, astrocytes and oligodendrocytes, i.e. all cell types populating the immature white matter. Assuming that the cingulate myelination deficit was associated with focal cingulate microglial activation in our model and that immature oligodendrocytes are highly vulnerable in an inflammatory context [27], [28], it is reasonable to hypothesize that melatonin could restore normal oligodendrocyte maturation by reducing white matter inflammation. In fact, in the present study, we found melatonin treatment to be associated with a reduction of microglial activation in GR pups at P3 *in vivo* and LPS-induced microglial activation *in vitro*. This result is in agreement with previous studies [29], [30]. The signalling pathways involved in the maturation effect of melatonin on developing oligodendrocytes remain unclear. However, several effects of melatonin through its receptors may account for its ability to prevent oligodendroglial damage: free radical scavenger production by activated microglia [17], [31], [32], improvement of membrane fluidity and reduction of edema and polymorphonuclear cell infiltration into damaged tissue, prevention of translocation of the nuclear factor-kappa-B to the nucleus and the subsequent reduction of pro-inflammatory cytokines expression, which play a relevant role in the inflammatory reaction [33], [34]. In addition, melatonin could modulate astrocyte reactivity or death through an upregulation of astrocytic anti-oxidative defenses [35], [36].

In conclusion, our results demonstrate that melatonin could preserve axonal myelination in adverse conditions. The neuroprotective pathway appears to implicate both melatonin receptors and inflammatory modulation, leading to a promotion of oligodendrocyte maturation. The present study also delineates the cellular mechanisms of melatonin neuroprotective benefits. Furthermore, our data strongly suggest that melatonin could be of great interest not only in perinatal white matter damage but also as a potential neuroprotective strategy for myelinopathy diseases observed in adults.

## Supporting Information

Figure S1 Double immunolabeling using GFAP (astrocytes in green, arrows) and Olig2 (oligodendrocytes in red, arrowheads) markers in cingulate white matter. Most of Olig2 nuclei did not colocalized with GFAP+ cells in the developing white matter.
(7.60 MB TIF)

Figure S2 Quantification of TUNEL+ cells detected in the hemispheric white matter at P3 from control (Ctl) and GR rat pups treated or not with Melatonin (Mel).
(2.36 MB TIF)

Figure S3 Quantitative analysis of the MBP-positive fibers optical density in the cingulate white matter of internal controls and sham control pups treated with either PBS or with melatonin 20 mg/kg.
(1.99 MB TIF)

Figure S4 Quantitative analysis of Ki67+ nuclei in the cingulate white matter according to the experimental groups at P3 and P14.
(1.83 MB TIF)

Figure S5 Immuno-labelling of primary oligodendroglial cell cultures using either APC or PLP at DIV6 and DIV10, respectively with or without treatment with 1 µmol melatonin.
(8.15 MB TIF)

Table S1 Brain weight (mean +/− SD) from delivery to P14 of rat pups in the experimental groups.
(0.03 MB DOC)
