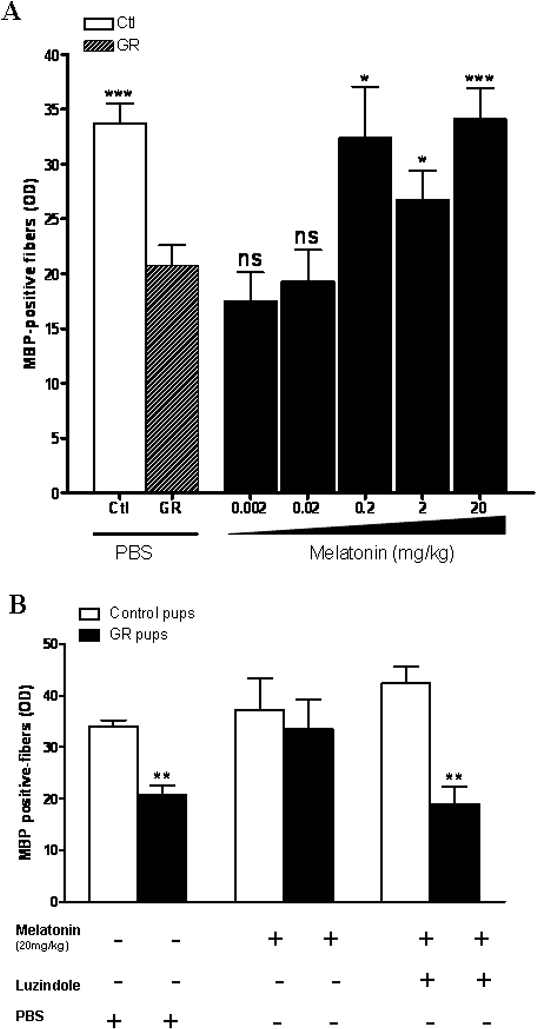# Correction: Melatonin Promotes Oligodendroglial Maturation of Injured White Matter in Neonatal Rats

**DOI:** 10.1371/annotation/d6a43156-441c-4dac-ab10-ee85b6215b1e

**Published:** 2009-10-15

**Authors:** Paul Olivier, Romain H. Fontaine, Gauthier Loron, Juliette Van Steenwinckel, Valérie Biran, Véronique Massonneau, Angela Kaindl, Jeremie Dalous, Christiane Charriaut-Marlangue, Marie-Stéphane Aigrot, Julien Pansiot, Catherine Verney, Pierre Gressens, Olivier Baud

There was an error in Figure 3. The corrected figure is available here: 

**Figure:**