# Supplementary figures and images for: Melatonin Promotes Oligodendroglial Maturation of Injured White Matter in Neonatal Rats
Source: PLoS One. 2009 Sep 22;4(9):e7128. doi: 10.1371/journal.pone.0007128 (PMC2742165; doi:10.1371/journal.pone.0007128)

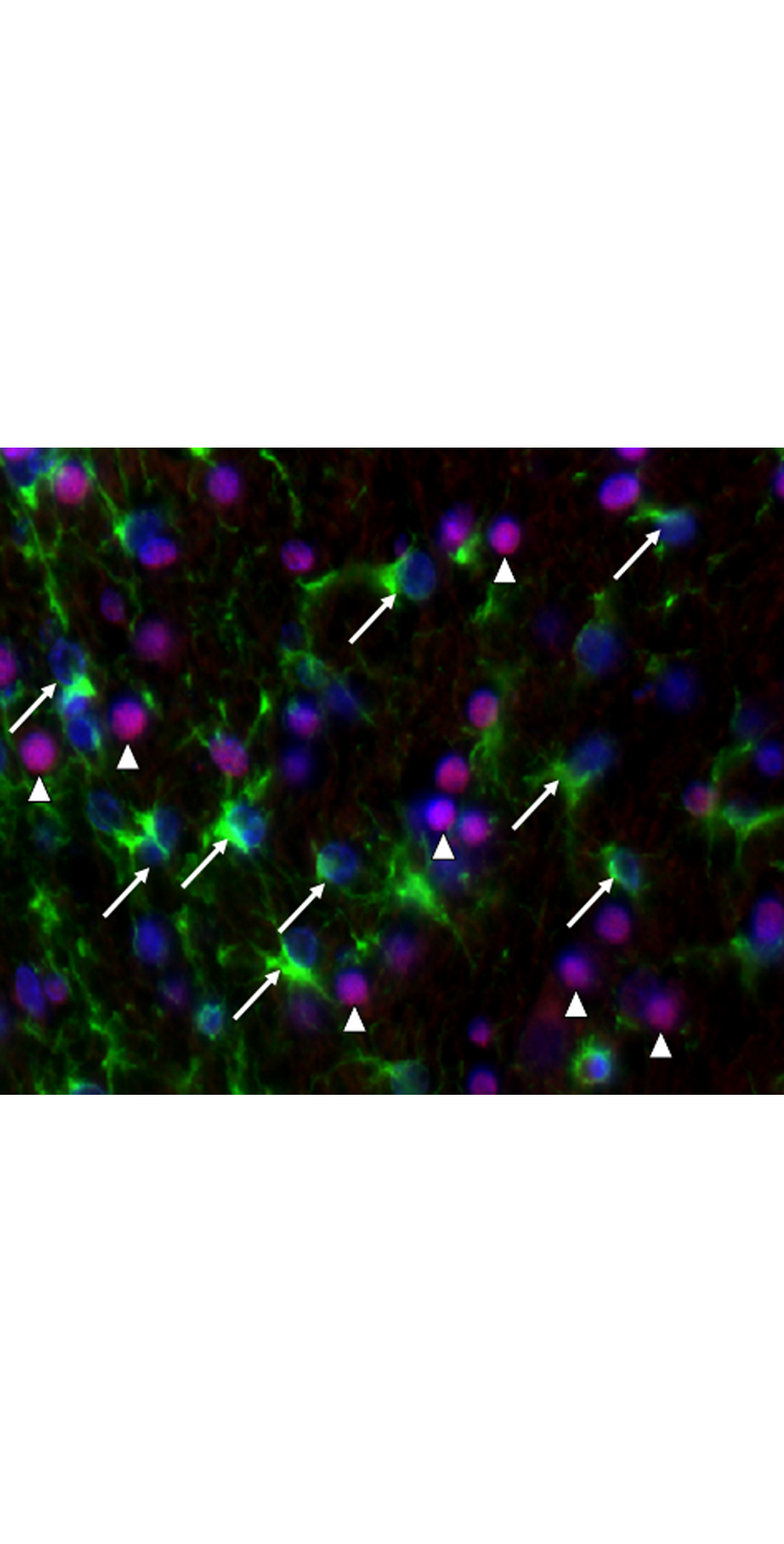

Supplement: Figure S1 — Double immunolabeling using GFAP (astrocytes in green, arrows) and Olig2 (oligodendrocytes in red, arrowheads) markers in cingulate white matter. Most of Olig2 nuclei did not colocalized with GFAP+ cells in the developing white matter. (7.60 MB TIF) [file pone.0007128.s001.tif]

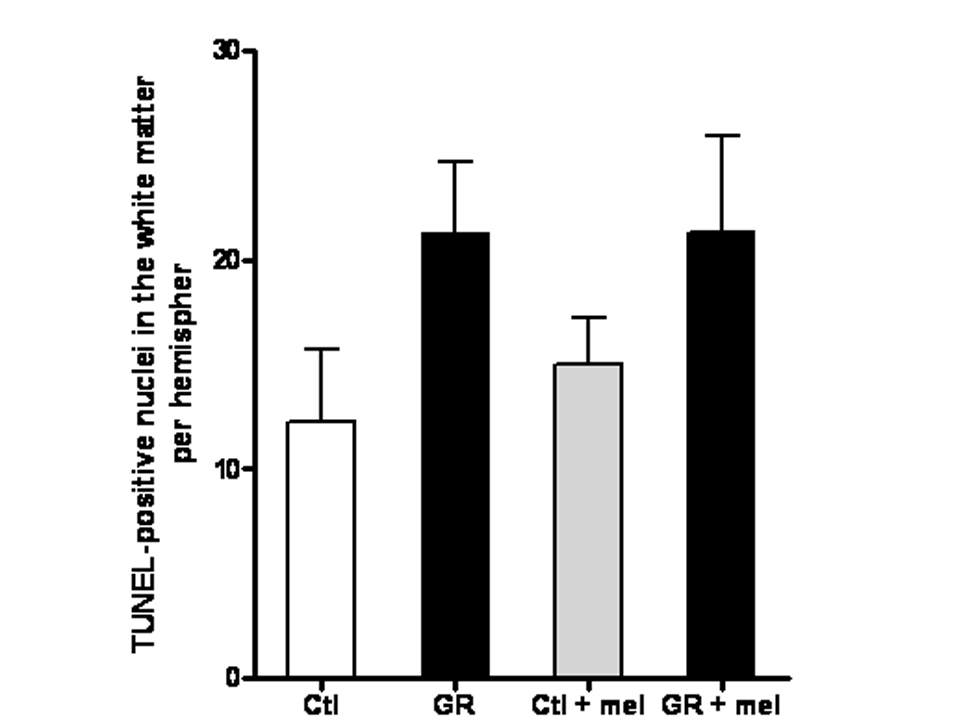

Supplement: Figure S2 — Quantification of TUNEL+ cells detected in the hemispheric white matter at P3 from control (Ctl) and GR rat pups treated or not with Melatonin (Mel). (2.36 MB TIF) [file pone.0007128.s002.tif]

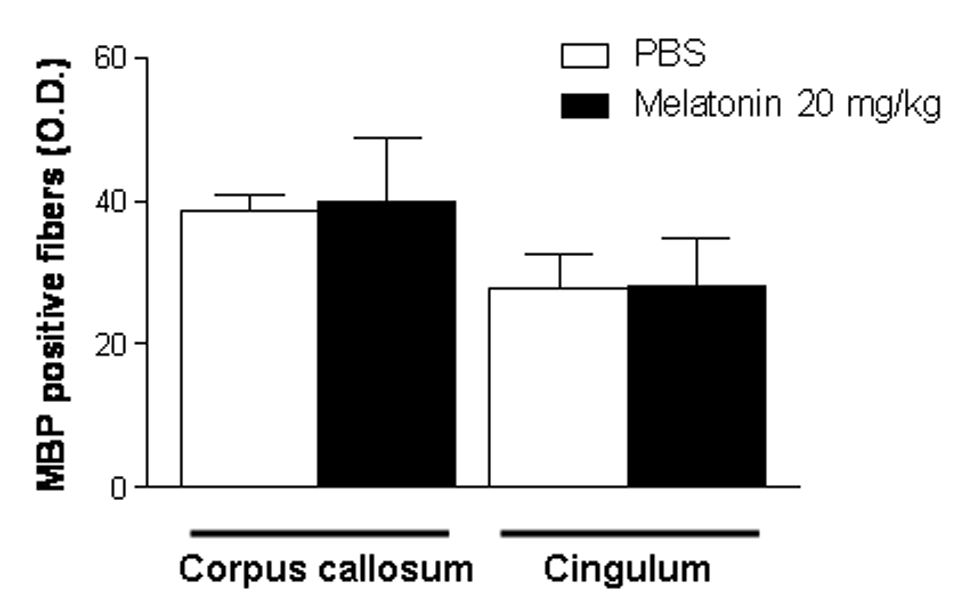

Supplement: Figure S3 — Quantitative analysis of the MBP-positive fibers optical density in the cingulate white matter of internal controls and sham control pups treated with either PBS or with melatonin 20 mg/kg. (1.99 MB TIF) [file pone.0007128.s003.tif]

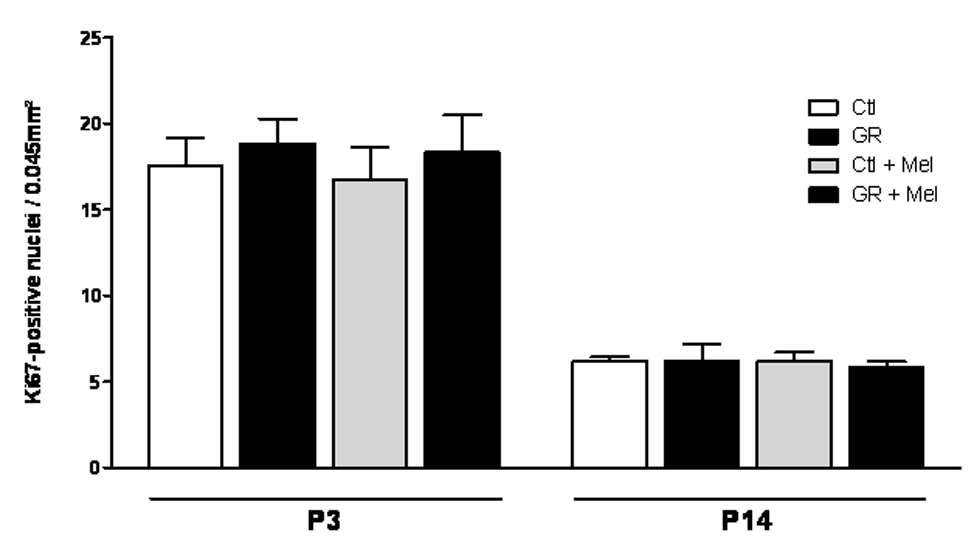

Supplement: Figure S4 — Quantitative analysis of Ki67+ nuclei in the cingulate white matter according to the experimental groups at P3 and P14. (1.83 MB TIF) [file pone.0007128.s004.tif]

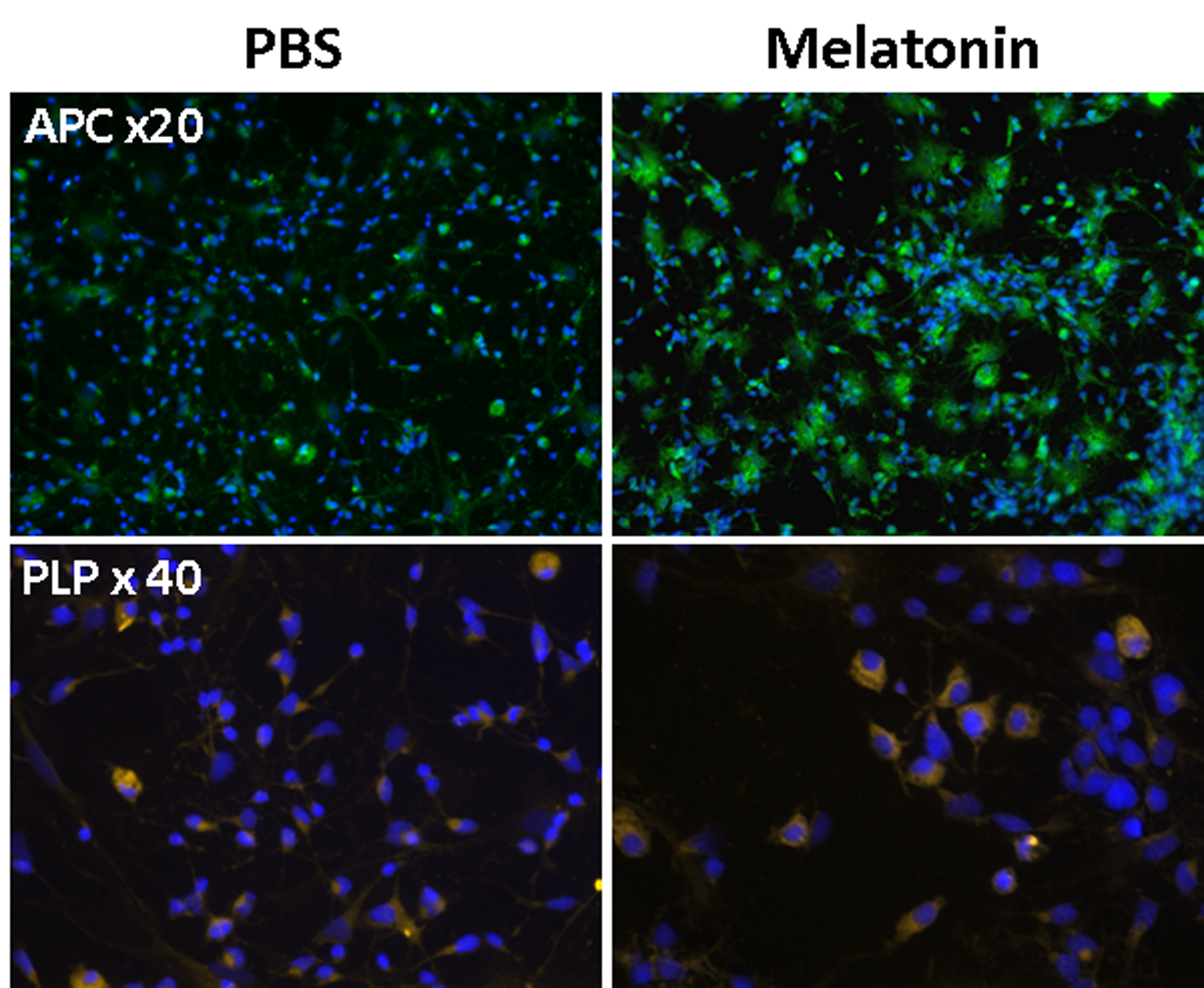

Supplement: Figure S5 — Immuno-labelling of primary oligodendroglial cell cultures using either APC or PLP at DIV6 and DIV10, respectively with or without treatment with 1 µmol melatonin. (8.15 MB TIF) [file pone.0007128.s005.tif]
