# Supplementary material for: Melatonin Promotes Oligodendroglial Maturation of Injured White Matter in Neonatal Rats
Source: PLoS One. 2009 Sep 22;4(9):e7128. doi: 10.1371/journal.pone.0007128 (PMC2742165; doi:10.1371/journal.pone.0007128)
Supplement: Table S1 — Brain weight (mean +/− SD) from delivery to P14 of rat pups in the experimental groups. (0.03 MB DOC) [file pone.0007128.s006.doc]

Supporting Table 1:

Brain weight (mean +/- SD) from delivery to P14 of rat pups in the experimental groups.

| **postnatal age of rat pups (day)** | | | | |
| --- | --- | --- | --- | --- |
| **Brain**  **Weight**  **(gm)** | **Group** | **P0** | **P3** | **P14** |
|  | Ctl pups | 0.22 ± 0.09  n = 12 | 0.34 ± 0.08  n = 8 | 1.08 ± 0.11  n = 10 |
|  | GR pups | 0.22 ± 0.03  n = 12 | 0.35 ± 0.08  n = 6 | 1.12 ± 0.15  n = 9 |
|  | Ctl + Mel | 0.20 ± 0.07  n = 14 | 0.41 ± 0.09  n = 7 | 1.11 ± 0.08  n = 15 |
|  | GR pups + Mel | 0.23 ± 0.02  n = 10 | 0.37 ± 0.08  n = 6 | 1.18± 0.19  n = 8 |
